# The Non-coding Side of Medulloblastoma

**DOI:** 10.3389/fcell.2020.00275

**Published:** 2020-05-27

**Authors:** Pietro Laneve, Elisa Caffarelli

**Affiliations:** Institute of Molecular Biology and Pathology, National Research Council, Rome, Italy

**Keywords:** non-coding RNA, microRNA, long non-coding RNA, neuronal differentiation, pediatric tumors, medulloblastoma, tumor subgroups

## Abstract

Medulloblastoma (MB) is the most common pediatric brain tumor and a primary cause of cancer-related death in children. Until a few years ago, only clinical and histological features were exploited for MB pathological classification and outcome prognosis. In the past decade, the advancement of high-throughput molecular analyses that integrate genetic, epigenetic, and expression data, together with the availability of increasing wealth of patient samples, revealed the existence of four molecularly distinct MB subgroups. Their further classification into 12 subtypes not only reduced the well-characterized intertumoral heterogeneity, but also provided new opportunities for the design of targets for precision oncology. Moreover, the identification of tumorigenic and self-renewing subpopulations of cancer stem cells in MB has increased our knowledge of its biology. Despite these advancements, the origin of MB is still debated, and its molecular bases are poorly characterized. A major goal in the field is to identify the key genes that drive tumor growth and the mechanisms through which they are able to promote tumorigenesis. So far, only protein-coding genes acting as oncogenic drivers have been characterized in each MB subgroup. The contribution of the non-coding side of the genome, which produces a plethora of transcripts that control fundamental biological processes, as the cell choice between proliferation and differentiation, is still unappreciated. This review wants to fill this major gap by summarizing the recent findings on the impact of non-coding RNAs in MB initiation and progression. Furthermore, their potential role as specific MB biomarkers and novel therapeutic targets is also highlighted.

## Introduction

Medulloblastoma (MB) is an aggressive tumor arising in the cerebellum, and one of the most frequent malignant central nervous system (CNS) cancers in childhood. Accounting for about 20% of all brain primary tumors in children younger than 14 years (Gajjar et al., 2004; Juraschka and Taylor, 2019), it represents one of the leading causes of pediatric tumor-related death with an overall annual incidence of about 5 cases per 1 million in the pediatric population (Siegel et al., 2013; Rusert et al., 2014; Huang et al., 2016; Ostrom et al., 2018). Medulloblastoma is considered a pediatric tumor since its incidence in adulthood is far rarer, with 0.05 cases per 100,000 population (Ostrom et al., 2018). Over the years, the consideration of this very heterogeneous tumor substantially changed, allowing the achievement of a better clinical risk stratification and therapeutic treatments, which have improved the overall survival rate of MB patients from 20% to approximately 80% in the last 35 years (Gottardo et al., 2014). This has been mainly due to the development of large-scale sequencing technologies that allowed the classification of MB into molecular subgroups and additional subtypes, each considered as a distinct disease. As a direct consequence, this categorization has opened the way to the development of molecular-based diagnoses and targeted therapeutic approaches. It is noteworthy that, so far, only protein-coding genes aberrantly expressed in distinct MB subgroups have been considered as potential tumor drivers, cancer biomarkers, and/or therapeutic targets. The implication of the predominant portion of the genome encoding for RNAs without coding potential, referred to as non-coding RNAs (ncRNAs), is only now coming into view.

## Non-Coding RNAs: Their Role in Physiology and Pathology

It is now well established that protein-coding genes represent only a small portion of the mammalian genome, the non-coding genes representing the vast majority. It has been established that transcription is pervasive, because more than 75% of the genome is transcribed (Djebali et al., 2012). The consequence is the production of a huge number of transcripts without coding potential, the ncRNAs. Initially regarded as transcriptional noise, ncRNAs were then reconsidered as crucial regulators of gene expression (The Encode Project Consortium, 2012). These findings, together with the discovery that the proportion of non-coding genes strongly increases with the eukaryotic complexity, suggest that in complex organisms the non-coding sequences contain a large amount of regulatory information, much of which is managed by RNA.

Several classes of ncRNAs have been described. They differ in length, structure, biogenesis and maturation, and in their mechanism of action (Laneve et al., 2019). Nevertheless, their common theme is the ability to regulate gene expression by sequestering from or delivering to specific targets other nucleic acids and/or protein factors. Through these mechanisms, ncRNAs control almost every step of gene expression, from epigenetic modifications on chromatin (Lee, 2012; Mercer and Mattick, 2013) to transcription and splicing in the nucleus and RNA stability (Faghihi and Wahlestedt, 2009) and translation (Huarte, 2013; Bartel, 2018) in the cytoplasm.

The first parameter used to classify ncRNAs was their nucleotide (nt) length. A threshold of 200 nt distinguishes long ncRNAs (lncRNAs) from short ncRNAs (Kapranov et al., 2007; Wang and Chang, 2011), which include several classes of transcripts, as the well characterized microRNAs (miRNAs) and the nuclear (snRNA) and nucleolar RNAs.

**FIGURE 1:**
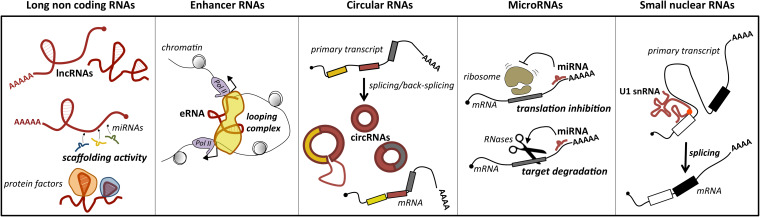
Main classes of ncRNAs implicated in MB. For each class, the predominant activity is depicted: lncRNAs act as scaffolds for microRNAs (ceRNA function) or protein factors, which affects their activity; enhancer RNAs (eRNAs) may have a pivotal role in promoting mRNA transcription by facilitating enhancer–promoter interaction; circular RNAs (circRNAs), for their biogenesis from a non-canonical splicing event, may have a role in the control of mature mRNA levels, or additional functions as miRNA or protein decoys; miRNAs by direct pairing with their mRNA targets trigger their translation inhibition or degradation; small nuclear RNAs, as components of the ribonucleoprotein machinery operating the splicing, may control this reaction underlying gene expression. The position of U1 snRNA mutation identified in MB is indicated by a red dot.

Long ncRNAs represent a loosely classified group of long transcripts that have recently attracted increasing attention for their unique versatility. Exploiting their large size, they may work as flexible modular scaffolds endowed with discrete domains for protein interactions and with sequences for selecting RNA and DNA targets (Figure 1). They comprise a heterogeneous class of transcripts, both intergenic and intragenic, as well as enhancer RNAs (eRNAs), which enhance the expression of their coding counterparts, and circular RNAs (circRNAs), covalently closed molecules derived from non-canonical splicing events (Memczak et al., 2013) (Figure 1). Long ncRNAs have been proposed to participate in relevant biological processes as proliferation, differentiation and development, by regulating gene expression at the epigenetic, transcriptional, or posttranscriptional levels. Furthermore, they may act *in cis*, affecting the expression of their nearby genes, or *in trans*, influencing genes very far away. In line with their function, genome-wide studies of tumor specimens have shown that a large number of lncRNAs are associated with various types of cancer and have established that their mutations, as well as their deregulated expression, promote tumor initiation and progression (Vitiello et al., 2015; Bhan et al., 2017). A major feature of lncRNAs is their tissue-specific expression, which is often higher than messenger RNAs (mRNAs; Djebali et al., 2012). In line with this, a growing body of evidence suggests that lncRNAs are promising candidates as diagnostic biomarkers or therapeutic targets for disease (Huarte, 2015).

MicroRNAs are a conserved class of endogenous small ncRNAs (20–23 nt long) that are processed from stem-loop regions of long transcripts. They mediate posttranscriptional control of gene expression, acting as negative regulators. In particular, they function as “guide RNAs” that deliver the silencing machinery to specific mRNA targets. By binding through base-pairing to response sequences generally distributed in the 3′ untranslated regions (3′ UTRs) of mRNAs, they promote degradation or translational repression of their target genes (Figure 1). Even if the effect of an individual miRNA on a target’s protein level is of fine-tuning, usually less than twofold (Baek et al., 2008), the combinatorial activity of different miRNAs on the same target strongly increases their repressive effect. Another salient feature of miRNAs is their pleiotropic activity. It means that an individual miRNA may target hundreds of target genes, which is relevant to canalize the regulatory programs. Through their activity, miRNAs influence gene programs underlying crucial biological processes as cell growth, proliferation, and differentiation, contributing to homeostasis and development (Jonas and Izaurralde, 2015; Bartel, 2018). In agreement with their biological importance, alterations in miRNA expression have been associated with tumorigenesis (Calin and Croce, 2006). Accordingly, miRNAs have been proposed as promising cancer biomarkers and potential therapeutic targets.

## Non-Coding RNAs and Cerebellum

Cerebellum has been considered for a long time as responsible for the acquisition of motor skills (Ramnani, 2006). However, over the past few decades, the advancements in neuroimaging studies, together with the development of computational model systems, have extended its contribution also to non-motor functions. Thanks to the connections with the cerebral cortex, the cerebellum is actively engaged in cognition and emotional activities (Ramnani, 2006; Koziol et al., 2014).

**FIGURE 2:**
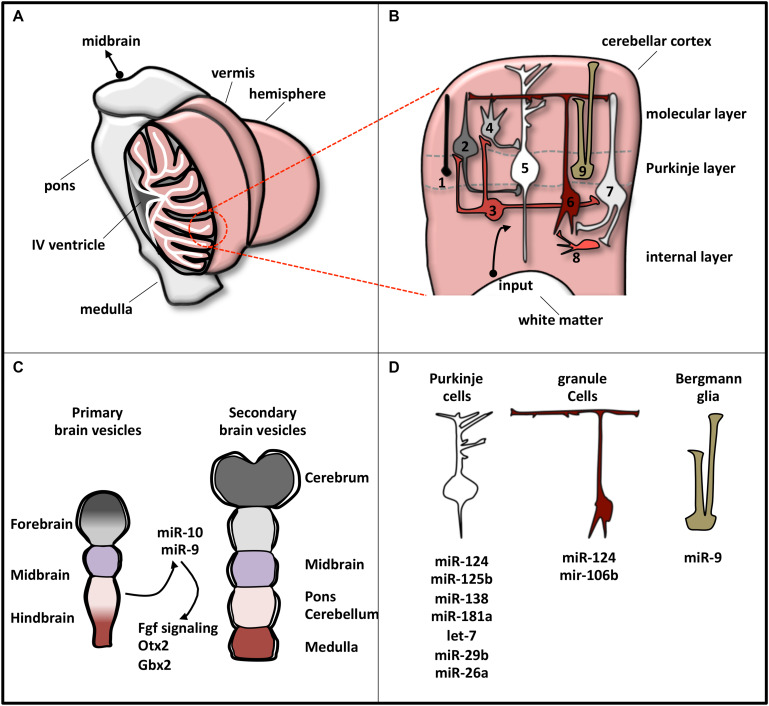
Schematic representation of cerebellar structure. **(A)** The cerebellum consists of two lateral hemispheres connected by a narrow midline area (vermis). In the picture, the left hemisphere is sectioned to show the IV ventricle (which separates the cerebellum from the pons) and the lobular structure of the cortex, made of convoluted folia of gray matter supported by branching central medulla of white matter. **(B)** Magnification of cerebellum cortex. The eight neuronal cerebellar cell types are numbered as follows: (1) candelabrum cells; (2) basket cells; (3) Lugaro cells; (4) stellate cells; (5) Purkinje cells; (6) granule cells; (7) Golgi cells; (8) unipolar brush cell. Bergmann glia is indicated as 9. These cell types compose the three layers, indicated on the right. Input pathways from white matter include mossy and climbing fibers. **(C)** Brain early embryonic development. Primary and secondary vesicles identify boundaries between the prospective brain regions. miR-9 and miR-10 are expressed in the cerebellar anlage and specify the midbrain–hindbrain boundary. **(D)** Specific cerebellar cells express a plethora of miRNAs that regulate their differentiation or function. The most representative miRNAs expressed in each cell type are reported.

It is a bilaterally symmetric structure originating from the dorsal part of the most anterior hindbrain, lying adjacent to the embryonic midbrain (Hibi and Shimizu, 2011; Butts et al., 2014). In human CNS, the cerebellum represents 10% of total brain mass and is the most architecturally complex region containing 80% of all neurons (Azevedo et al., 2009; Butts et al., 2012). It can be divided, along the mediolateral axis, into a midline vermis and two lateral hemispheres (Figure 2A; Constantin, 2017).

At a variance with this complexity, cerebellum is a very simple structure from a histological point of view (Figure 2B). Cerebellar cortex contains eight neuronal populations that are organized in three layers. The intermediate layer (Purkinje cell layer) contains the soma of Purkinje cells and candelabrum cells and is squeezed between a superficial (molecular) layer, containing a network of neuronal processes and interneurons, and an internal (granular) layer, composed of granule cells, Lugaro cells, interneurons, and unipolar brush cells (Constantin, 2017). Additionally, inside the Purkinje cell layer, a population of unipolar astrocyte, the Bergmann glia, is present, extending its radial fibers into the superficial layer.

In humans, cerebellar development occurs in the third trimester and continues beyond birth. During development, its surface increases significantly because of the formation of lobules allocating in a small area the large number of neurons, mainly granule cells (Scott et al., 2012). The territory from which the cerebellum originates is close to the hindbrain–midbrain boundary (the “isthmus”), an organizing center in the vertebrate neural tube required for the development of the midbrain–hindbrain domain. Recently, the involvement of miRNAs in the regulatory circuits that guarantee the establishment and the maintenance of hindbrain–midbrain boundary has been highlighted (Figure 2C). Mir-10 has been shown to downregulate key midbrain markers as *Otx2* and to upregulate hindbrain markers caudal to mid–hindbrain boundary as *Gbx2* in human neural progenitor cells (NPCs) (Jonsson et al., 2015). Loss of miR-10 expands the Gbx2 domain affecting the cerebellar development (Katahira et al., 2000). In zebrafish, miR-9, which is expressed adjacent to the midbrain–hindbrain boundary, reduced the boundary size by targeting components of the Fgf signaling pathway (Leucht et al., 2008), whereas in frog it promotes neurogenesis in the hindbrain by modifying the onset of the antineurogenic bHLH transcription factor (TF) program (Bonev et al., 2011).

MicroRNA profile analyses, knockout of the miRNA biosynthetic factor Dicer, and specific miRNA manipulations have revealed the pattern of miRNA expression in individual cerebellar cell types and have clarified miRNA implication in cell development or function (Figure 2D). In Bergmann glia, miRNAs, among which miR-9, establish specific transcriptional signatures ensuring proper cerebellar morphology (Tao et al., 2011; Kuang et al., 2012). In Purkinje cells, the expressed miRNAs (Pieczora et al., 2017) protect neurons from degeneration (Schaefer et al., 2007). Finally, in granule cells, Dicer-dependent pathways sustain cell development through the SHH signaling (Constantin and Wainwright, 2015) and the DNA damage response (Swahari et al., 2016).

## Medulloblastoma: From the Initial Discovery to the Present Classification

In 1910, James Homer Wright described for the first time MB as a distinct CNS tumor and proposed that it may derive from restricted neuronal precursor cells, referred to as “neuroblasts” (Wright, 1910). Later, in 1926, Bailey and Cushing (1926) formulated a new theory on the origin of MB. They postulated that it is a posterior fossa brain tumor derived from primitive embryonic neuroepithelial cells, termed “medulloblasts,” that reside in the primitive neural tube (Rutka and Hoffman, 1996; Kunschner, 2002; Millard and De Braganca, 2016). Taking advantage of his activity as a neurosurgeon, Cushing described the salient features of MB as a disease occurring mainly in preadolescents, with a relatively short history of symptoms and signs and the tendency to originate from the cerebellum vermis (Cushing, 1930). A further step toward the comprehension of the disease occurred in 1973, when MB was classified as a primitive neuroectodermal tumor for its histological features. At the same time, its origin from undifferentiated cells in the subependymal zone was hypothesized (Hart and Earle, 1973). However, only with the rise of the molecular era this hypothesis was overtaken, and MB started to be considered as a molecularly distinct brain tumor, arising from cerebellar granule cells (Pomeroy et al., 2002).

In 2007, the World Health Organization (WHO) published a classification of CNS tumors, primarily based on histopathological features. Accordingly, MBs were assigned to one of the four entities: classic (CMB), desmoplastic/nodular (DNMB), extensive nodular (MBEN), or anaplastic/large cell (LC/A) MB (Louis et al., 2007). The CMB variant is the most common histological subtype and is characterized by prototypical sheets of repetitive small cells with round nuclei and a high nuclear-to-cytoplasmic ratio (Northcott et al., 2012a). The MBEN variant occurs predominantly in infants. It is related to DNMB but differs for the presence of a markedly expanded lobular architecture, due to the presence of large zones, rich in neuropil-like tissues, containing a population of small cells similar to those of a central neurocytoma. A further difference between MBEN and DNMB relates to the internodular reticulin-rich component, which is reduced in MBEN, whereas it dominates the DNMB variant (Louis et al., 2007). The LC/A type displays high levels of atypia and is characterized by marked nuclear pleomorphism, cell–cell wrapping, and high mitotic activity. This variant shows cytological overlap with the highly malignant LC MB, which is characterized by spherical cells, open chromatin, and prominent central nucleoli (Louis et al., 2007).

Over the past two decades, the rapid advances in genomics, epigenomics, and transcriptomics studies have tremendously accelerated the process of identification of genes, pathways, and biological processes underpinning MB onset. The -omics analyses were conducted by several international consortia, as the International Cancer Genome Consortium (Jones et al., 2012; Rausch et al., 2012; Kool et al., 2014; Northcott et al., 2014), the Pediatric Cancer Genome Project (Robinson et al., 2012), and the Medulloblastoma Advanced Genomics Consortium (Northcott et al., 2012c; Morrissy et al., 2016), and led to a new interpretation of MB as a collection of distinct diseases.

Genomics data, through the identification of frequently mutated new cancer genes, revealed the occurrence of distinct MB subgroups. Epigenomics and transcriptomics approaches in the postgenomic era disclosed typical epigenetic and transcriptional signatures that definitely converged on four MB subgroups. They are the better described Wingless (WNT) and Sonic Hedgehog (SHH) subgroups and the less characterized groups 3 and 4 (Northcott et al., 2011a, b, 2012a; Taylor et al., 2012). These new MB entities, allowing the shift from a tumor classification based on purely histological parameters to a new molecularly oriented categorization, were incorporated into the current 2016 WHO Classification of CNS tumors (Louis et al., 2016).

Recently, further studies investigated the proteomic landscape of MBs through quantitative mass spectrometry carried out on primary tumors (Rivero-Hinojosa et al., 2018). These analyses, while confirming the classification of MBs into the consensus subgroups, revealed a poor correlation between mRNA and protein expression, highlighting the crucial role of posttranscriptional mechanisms in MB etiology.

Moreover, studies making use of DNA methylation genome-wide approach, carried out on a large cohort of primary MB samples, produced new data that were integrated with gene expression profiles. These combined efforts provided a more comprehensive view of MB, further refining the molecular and clinical heterogeneity. Exploiting the similarity network fusion, a method of integrative clustering of multiple heterogeneous data sources (Wang et al., 2014), 12 new MB subtypes have been described. Even if not yet deeply characterized, the MB subtypes display distinct somatic copy-number aberrations, specific transcriptional signatures, differentially activated pathways, and different clinical outcomes (Cavalli et al., 2017). This important achievement will be helpful to resolve the molecular mechanisms and oncogenic drivers underlying the etiology of each subtype. This, in turn, will eventually shape driver events that are typical of a specific subtype, even if uncommon at a subgroup level.

### WNT Subgroup and Subtypes

Among MBs, the best known subgroup is WNT, which occurs primarily in children from 4 years to early adulthood (Kool et al., 2012; Northcott et al., 2012b; Hovestadt et al., 2020) and accounts for about 10% of all MBs. WNT-MBs are usually of classic histology, are associated with most favorable prognosis, with 95% survival at 5 years in pediatric patients, are rarely metastatic at diagnosis (5–10% of cases), and rarely recur (Juraschka and Taylor, 2019; Hovestadt et al., 2020). WNT tumors are typically located midline with involvement of the brainstem or are positioned in the cerebellar peduncle and cerebellopontine angle cistern (Perreault et al., 2014). The hallmark feature of WNT-driven MB, found in about 85% of patients, are somatic mutations in *Ctnnb1* gene, which encodes β-catenin. The increased stability of the β-catenin protein induces the constitutive activation of WNT pathway and the consequent activation of WNT-responsive genes that promote cell proliferation (Northcott et al., 2017; Wang et al., 2018). The majority of WNT tumors lacking *Ctnnb1* mutations contain mutations in the *Apc* tumor-suppressor gene (Taylor et al., 2012; Waszak et al., 2018). Other genes recurrently mutated in these tumors are *Ddx3x* (36%), encoding a putative RNA helicase involved in chromosome segregation and cell cycle progression (Jones et al., 2012; Pugh et al., 2012; Robinson et al., 2012), and the genes encoding epigenetic factors as S*marca4* (19%) and C*rebbp*, suggesting that deregulation of the epigenome may be relevant for MB tumorigenesis (Robinson et al., 2012). This subgroup rarely displays copy number aberration except for loss of one copy of chromosome 6 (monosomy 6) occurring in 86% of patients (Northcott et al., 2012c, 2017).

The existence of two WNT subtypes—WNTα and WNTβ—has also been described (Cavalli et al., 2017). They differ in several features: WNTα occurs in 70% of cases, primarily affects children (median age of 10 years), and is characterized by monosomy 6. WNTβ has a lower incidence (30% of cases) and occurs in older children and adults, who infrequently have monosomy 6.

### SHH Subgroup and Subtypes

Approximately 30% of all MBs are classified as SHH tumors. They display CMB and DNMB histologies occurring at similar frequency (by about 40%), whereas the remaining cases are of LC/A histology (Hovestadt et al., 2020). SHH-MBs are characterized by an intermediate prognosis with overall survival rates ranging from 60 to 80% (Cho et al., 2011; Northcott et al., 2011a, b; Kool et al., 2012; Taylor et al., 2012). SHH-MBs typically arise in the cerebellar hemispheres, and most often occur in infants and adults, with a minority of cases described in childhood (Perreault et al., 2014). Typical alterations of this subgroup include germline or somatic mutations or copy number alterations of components of the SHH pathway that results constitutively activated. Among mutated or deleted genes, there are *Ptch1* (43%) and *Sufu* (10%), encoding negative regulators of the SHH pathway (Johnson et al., 1996; Taylor et al., 2002; Brugières et al., 2010). Amplifications of SHH target genes as *MycN* (7%) and *Gli1* or *Gli2* (9%) (Gibson et al., 2010; Perreault et al., 2014; Raybaud et al., 2015) are frequently observed.

Other recurrent mutations, occurring in 30% of childhood SHH-MB, concern the *Tp53* gene and are associated with poor outcomes (Zhukova et al., 2013; Louis et al., 2016; Ramaswamy et al., 2016). Differently from WNT subgroup, frequent cytogenetic events in SHH tumors include loss of chromosome 9q (causing loss of heterozygosity of *Pitch1*) and 10q (loss of *Sufu*) (Northcott et al., 2017).

Four subtypes, SHHα, SHHβ, SHHγ, and SHHδ have been described (Cavalli et al., 2017). SHHα tumors primarily affect children aged 3–16 years, have the worst prognosis, and are characterized by frequent *Tp53* mutations and *Myc/Gli2* amplifications. SHHβ tumors occur in infants and are frequently metastatic; they harbor focal *Pten* deletions (25% of cases) and have multiple focal amplifications. SHHγ occurs in infants and displays extensive nodularity histology. The SHHδ subtype, typical of adulthood, is enriched for *Tert* promoter mutations.

### Group 3 Subgroup and Subtypes

Group 3 MB occurs almost exclusively in infants and young children, with a male predominance and a prevalent LC/A histology. Anatomically, these tumors have a midline vermian location adjacent to the fourth ventricle (Taylor et al., 2012; Perreault et al., 2014; Hovestadt et al., 2020). Group 3 accounts for approximately 25% of all MBs and is the most aggressive of the four subgroups, with the worst survival outcomes (<60% at 5 years) and the highest rates of metastasis at diagnosis (40–45%). Nevertheless, it is still considered an enigmatic tumor, because a common driver pathway has not yet been identified (Taylor et al., 2012). The most common genetic aberration is the amplification of *MYC* oncogene (17–20% of patients), which represents the major group 3 signature. *MYC* amplification frequently co-occurs with *PVT1-MYC* fusion, where PVT1 is a lncRNA supposed to stabilize the MYC protein (Northcott et al., 2012c; Tseng et al., 2014).

Gene mutations are rare in this group, and only four genes, namely, *Smarca4*, *Kbtbd4*, *Ctdneo1*, and *Kmt2d*, are mutated in 5% of cases (Northcott et al., 2017). Small fraction of group 3 tumors is associated with amplification of *Mycn* (5%) and of the TF *Otx2* (3%) (Northcott et al., 2017). Also, enhancer activation of *Gfi1* and *Gfi1b* expression has been observed in 40–50% of cases (Northcott et al., 2014). Moreover, this subgroup is characterized by genomic instability with gains of chromosome 1q,7 and deletions of 10q, 11, 16q, and 17p (Northcott et al., 2012a).

Two subtype classifications have been proposed for group 3 MB. The first one is based on methylation data and identifies as an high-risk subtype that displaying *Myc* amplification and a hypomethylation phenotype (Schwalbe et al., 2017). The second classification identifies three subtypes: 3α, occurring in infants, frequently metastatic but associated with a favorable prognosis; 3β, displaying a high frequency of activation of the oncogenes *Gfi1* and *Gfi1b* and of *Otx2* and rarely metastatic; and 3γ occurring in infants, associated with *Myc* amplification and displaying the worst prognosis (Cavalli et al., 2017).

### Group 4 Subgroup and Subtypes

Group 4 is the least understood and the most common subgroup among MBs. It accounts for 35–40% of all MB diagnoses and typically occurs in childhood and adolescence with a higher frequency in males (3:1 sex ratio) (Taylor et al., 2012). The outcome of group 4 patients is intermediate even if metastases are often present at diagnosis. In approximately 6–9% of cases, common mutations in *Kdm6a*, *Zmym3*, *Ktm2c*, and *Kbtbd4* genes have been described, together with the amplification of *Mycn* and *Cdk6* genes and the overexpression of *Prdm6* gene, which is frequently associated with *Sncaip* duplication events (Northcott et al., 2017). Group 4 MB has been further subdivided into three subtypes: group 4α characterized by *MycN* and *Cdk6* amplifications and strongly enriched for 8p loss, group 4β displaying *Sncaip* duplication, and group 4γ enriched for focal *Cdk6* amplification and for 8p loss (Cavalli et al., 2017).

## Medulloblastoma Origin

Besides the genome, epigenome, and transcriptome alterations, the heterogeneity of the four MB subgroups may be partially due to their different developmental origins. Identification of the specific cell types these tumors originate from may be very informative for both the understanding of the malignancy and the development of appropriate treatments. Medulloblastoma tumors are thought to originate in the cerebellum, except for the WNT subgroups that arise outside the cerebellum and are distributed within the fourth ventricle and infiltrated the dorsal surface of the brainstem (Gibson et al., 2010). Dorsal brainstem progenitor cells of cochlear, mossy fiber, and climbing fiber neurons are regarded as the potential source of these tumors (Gibson et al., 2010), following the activation of *Ctnnb1* and the concurrent *Trp53* deletion (Lu et al., 2019).

Differently, all available SHH-subtype tumors were localized away from the brainstem, within the cerebellar hemispheres. They are thought to originate from cerebellar neural stem cells (NSCs) or committed granule neuron precursor cells (GNPCs) following aberrant activation of SHH (Gibson et al., 2010; Lu et al., 2019). The group 3 MB tumors are often positioned near the fourth ventricle, pointing to cerebellar stem/progenitor cells or GNPCs as potential sources (Kawauchi et al., 2012; Pei et al., 2012; Lu et al., 2019). The source of group 4 MB is still debated. Based on the spatiotemporal activity of a subset of group 4 master TFs, deep cerebellar nuclei, residing in the cerebellar nuclear transitory zone, or their earlier precursors deriving from the upper rhombic lip, are considered their putative cells of origin (Lin et al., 2016).

A major shift in our understanding of MB origin occurred very recently, thanks to the use of large-scale single-cell RNA sequencing (RNA-Seq), a powerful technique to identify cellular populations that are biologically distinct on the basis of gene activity. This transcriptome analysis, carried out on RNA from murine cerebellum at specific time points of development, allowed the identification of specific neural cell types and subtypes and their anatomical location and putative developmental origin, and to draw pseudotime trajectories for the various cerebellum lineages (Carter et al., 2018; Vladiou et al., 2019). By applying single-cell analysis on a cohort of primary MBs, two independent research groups revealed the high transcriptional heterogeneity of MB subgroups (Hovestadt et al., 2019; Vladiou et al., 2019).

Following comparison between the transcriptomes of lineage-restricted cell populations during cerebellum development and of MB cells, Vladiou et al. (2019) were able to demonstrate that SHH-MB includes a variety of cell types with various levels of differentiation and growth capacity mirroring the temporal evolution of the developing GNPC hierarchy. In particular, SHH-MBs better match to the GNPCs in the early postnatal period. RNA-Seq from Group 3 MB revealed the presence of highly divergent lines of differentiation that mirror normal development along the GNPC, unipolar Brusch cell, Purkinje cell, and GABAergic interneuron lineages. This indicates an origin from uncommitted cerebellar stem cells, followed by differentiation of transformed cells along diverse developmental lineages. The same analysis carried out on group 4, whose cell of origin is unknown, revealed that these tumors display a better match with the transcriptomes of the UBC lineage at several time points during UBC development. However, the observation that group 4 MB cells simultaneously express both GNPC and UBC marker genes suggests that group 4 arises from a population of bipotential progenitor cells that are able to generate cells of both the GNPC and UBC lineages.

## MB Cancer Stem Cells

An additional cause of intratumor heterogeneity is ascribed to cancer stem cells (CSCs), which are responsible for triggering tumor initiation, maintenance, and progression *in vivo.* Cancer stem cells result from the accumulation of transforming mutations and maintain two abilities: self-renewal, which allows the expansion of CSC pool, and differentiation, through which they generate the heterogeneous cell lineages that constitute the bulk of the tumor (Huang et al., 2016).

The CSC model, proposed to explain the intratumor heterogeneity, derives from studies carried out on different tumors including brain tumors (Singh et al., 2003). Medulloblastoma stem cells (MBSCs) have been initially identified and sorted using specific stem cell biomarkers, as CD133 and CD15, and different approaches, such as flow cytometry, xenograft models, and lineage tracing. The first studies revealed that CD133^+^/Nestin^+^ cells isolated from MB tissue were able to proliferate, self-renew, and differentiate *in vitro* (Singh et al., 2003). However, further studies concluded that also CD133^–^ MB cells possessed the same properties (Srivastava and Nalbantoglu, 2008; Read et al., 2009), highlighting the troubles in isolating MBSCs. A significant step forward in the identification of MBSCs was done exploiting the genetically engineered mouse (GEM) models established on the basis of MB molecular classification. In 2009, a tumor-propagating CD15^+^ cell was isolated from a *Patched* haploinsufficient (*Ptch*^+/–^) MB model (Read et al., 2009). These cells showed stem-like and tumor-initiating capacity (Ward et al., 2009). It was also demonstrated that CD15^+^ MBSCs express aberrant levels of the SHH target genes *Gli1* and *CyclinD1*, which in turn suggests that their increased proliferative capacity is related with increased activation of SHH pathway (Vanner et al., 2014). In addition, the findings that Gli1/2, the main effectors of the SHH pathway, interact with the stemness factors Nanog (Po et al., 2010) and MycN (Marino, 2005) and with the polycomb protein Bmi-1 (Leung et al., 2004; Wang et al., 2012) in the self-renewal regulation of MBSCs indicate that the SHH pathway has a pivotal role in MBSC maintenance and activity. A role in MBSC self-renewal has also been unveiled for the Notch pathway that is involved in the regulation of stem cells both in physiological and pathological conditions. In particular, the main effector of the pathway Hes1 was found upregulated in CD133^+^ MB cells, and its downregulation largely reduced the CD133^+^ cell fraction (Fan et al., 2006).

More recently, it was shown that quiescent Sox2^+^ MB cells from postirradiated *Ptch*^+/–^ mice were tumorigenic and showed a greater self-renewal capacity than their proliferating progeny (Vanner et al., 2014). Further studies revealed a tight association of oncogenes as *Myc* (Venkataraman et al., 2014) and *MycN* (Ahmad et al., 2015), which are aberrantly amplified in MB, with MBSC stemness. Accordingly, inhibition of Myc-regulated transcription program causes the suppression of stem cell–associated signaling in MB cells and the inhibition of MB tumor cell self-renewal (Venkataraman et al., 2014). Similarly, it was shown that depletion of *MycN* in tumor-derived neurosphere cell line, derived from a GEM model of MycN-driven MB, negatively affects the expansion of cells expressing markers of NSCs and/or progenitors associated with MB tumorigenesis (Ahmad et al., 2015).

Increasing evidence indicates that also the non-coding portion of the genome participates in the regulation of cancer cell stemness and in the maintenance of CSC population (see section “MiRNAs and MB Cancer Stem Cells”).

## MiRNAs in MB

A number of studies aimed to identify miRNAs engaged in MB tumorigenesis, in order to define novel markers for accurate diagnosis and regulatory modules as prospective targets for therapeutic interventions. As a consequence, miRNAs have been largely associated with MB, in which their aberrant expression or mutations (Lu et al., 2009; Lv et al., 2012) underpin oncogenic or oncosuppressive functions.

These research lines took advantage of high-throughput methods, candidate-oriented approaches, or even combined strategies.

### High-Throughput Analyses of miRNAs in MB: Pioneering Studies

To globally investigate the involvement of miRNAs in MB carcinogenesis, Ferretti et al. (2009) performed the first high-throughput miRNA expression profile in human MB specimens. This study: (1) highlighted that miRNAs were predominantly downregulated in MB, suggesting a general function as tumor suppressors; (2) allowed the identification of specific miRNA signatures, which distinguished tumors from healthy tissues, which recognized distinct MB histotypes or subsets, and which correlated with disease risk and (3) identified single miRNA candidates for functional analyses. Among them, miR-9 and miR-125a, previously shown to inhibit proliferation of cells of neuroblastoma (NB) (Laneve et al., 2007), a pediatric tumor of the sympathetic NS, were confirmed to arrest MB cell growth, through both reduction of cell proliferation and increase of apoptosis. MiR-9 and miR-125a: (1) target the truncated isoform of the tyrosine kinase receptor C (tTRKC), overexpressed in many tumors; (2) balance the ratio with the full-length isoform (Kim et al., 1999; Grotzer et al., 2000) and (3) are associated with a favorable prognosis. This research group also analyzed the role of miRNAs in SHH pathway, whose constitutive activation makes GNPCs susceptible of malignant transformation into MB (Ruiz et al., 2002; Kimura et al., 2005). MicroRNA expression profiling was performed in two subsets of human primary MBs (Ferretti et al., 2008), showing high or low SHH signaling strength (i.e., high or low Gli levels). A signature of 30 downregulated miRNAs was identified in Gli^High^ tumors, suggesting that loss of specific miRNAs may be associated with SHH signaling alteration in MB. Among the deregulated species, miR-125b, miR-326, and miR-324-5p were found to control the expression of positive members of the pathway, the activator Smo, and the effector Gli1. This miRNA-mediated circuitry antagonizes SHH activity during cerebellar GNPC differentiation, whereas its abrogation during neuronal development promotes brain tumorigenesis. Interestingly, the loss of miR-324-5p is caused by chromosome 17p deletion, a hallmark of approximately 40% of MBs. Later on, the same authors demonstrated that miR-326 and its host gene Arrb1, encoding for an adaptor and scaffold protein regulating several signaling pathways involved in cell development and cancer (DeWire et al., 2007), are both downregulated in MBSCs derived from SHH-MB, where they act as negative regulators of self-renewal at the posttranscriptional and posttranslational level (Miele et al., 2017).

Finally, Birks et al. (2011) were among the first to apply microarray-based determination of miRNA expression to pediatrics brain cancers, among which MB. Interestingly, besides specific signatures of overexpressed or downregulated miRNAs in each tumor group, differential expression of miR-129, miR-142-5p, and miR-25 in all tumor types, compared to normal tissues, was unveiled.

### MiRNA Profiling as a Tool for MB Diagnosis and Stratification

In the following decade, great emphasis was devoted to the study of small regulatory RNAs. MicroRNA profiling was largely exploited to identify useful biomarkers for MB diagnosis (Dai et al., 2017; Tantawy et al., 2018), classification, and clinical management.

The detection of miRNAs in biological liquids (Chen et al., 2008; Mitchell et al., 2008), including cerebrospinal fluid (CSF) (Baraniskin et al., 2011; Teplyuk et al., 2012; Shalaby and Grotzer, 2015), coupled to miRNA high stability and easy detectability, makes them ideal diagnostic and prognostic markers for brain tumors. Croce’s group utilized for the first time NanoString, a variation of microarray technology, to identify a CSF miRNA signature that distinguishes among CNS malignancies (Drusco et al., 2015). More than 80 samples were collected from 34 patients with CNS benign and malignant tumors, including MB. MiR-451, -711, -935, -223, and -125b were found differentially expressed between and among groups. These miRNAs were validated, by reverse transcriptase–polymerase chain reaction (RT-PCR) and *in situ* hybridization, as promising cancer CSF markers for accurate diagnosis of CNS neoplasms. The identification of candidate circulating biomarkers remains a primary goal in cancer research. Analysis of the proteome and miRNome of extracellular microvesicles (MVs), released by highly aggressive stem-like MB cells, detected 10 miRNAs exclusively present in MVs of cells overexpressing the pluripotent factor *Oct4A* and revealed ERK, PI3K/AKT/mTOR, and EGF/EGFR as the primary altered oncogenic signaling pathways in these cells (Kaid et al., 2020).

Other studies used extensive miRNA analysis for classifying primary MBs (Cho et al., 2011), also in the rare adult cases (Kaur et al., 2016). By low-density array, Gokhale et al. (2010) identified 216 MB-expressed miRNAs that segregated into subgroups closely matching those identified by protein-coding gene profiling. The most robust miRNA signature was found in WNT-MB. Among the 16 differentially expressed miRNAs in the WNT subgroup, the two most upregulated species, miR-193a and miR-224, were functionally validated in MB cell lines. They were able to reduce proliferation, to increase radiation sensitivity, and to inhibit anchorage-independent cell growth, indicating their contribution to the lower metastatic potential and better response to radiation therapy of WNT-MB. In 2013, the same authors confirmed the effectiveness of miRNA-based molecular subgrouping on additional 103 MBs, including 59 formalin-fixed paraffin-embedded tissues (Kunder et al., 2013). Later on, miR-449a (Li et al., 2016) and miR-148a (Yogi et al., 2015) were reported as candidate tumor-suppressor genes and potential diagnostic markers or therapeutic targets in WNT-MBs.

Large-scale miRNA evaluations also allowed inferring MB bioclinical features (Pezuk et al., 2017). MicroRNA microarrays in pediatric samples of CNS neoplasms (MB and atypical teratoid/rhabdoid tumors), together with meta-analyses based on available miRNA datasets, identified and validated miRNA candidates whose differential expression was associated with MB prognosis: age at diagnosis, disease progression, and clinical outcome (Braoudaki et al., 2014). In particular, miR-34a was found as upregulated in all the samples tested.

Recently, high-throughput sequencing on tissues from different subgroups of MB revealed miRNA signatures distinguishing between groups 3 and 4 (Gershanov et al., 2018), whose discrimination is still challenging due to the paucity of available biomarkers. Of 783 expressed miRNAs in at least 1 sample, 462 were common to all subgroups, and 19 were differentially expressed between group 4 and the others. Three miRNAs, miR-20a-5p, 181a-2-3p, and 224-5p, were identified as specific group 4 markers, able to distinguish between group 4 and group 3. Integrated miRNA–mRNA analysis revealed the anticorrelation between group 4 differentially expressed miRNAs and their predicted/validated mRNA targets, identifying at least five inversely expressed miRNAs–mRNAs couples involved in pathways as cellular development, cellular growth and proliferation, and connective tissue development and function.

Taking advantage of integrated analyses downstream to comprehensive RNA profiling in primary MBs, additional reports highlighted the miRNA–mRNA anticorrelation in MB, revealing coding and ncRNA pathways possibly relevant in this malignancy (Kumar et al., 2018). Overall, these findings support the potential of miRNAs as biomarkers for MB classification. In addition, the identification of miRNA–mRNA regulatory networks underscores specific genes or gene pathways as candidate targets for MB molecular therapies.

### MiRNA and mRNA Integrated Analyses in MB Progression

The categorization of MB into distinct subgroups with specific mutational and expression profiles fueled miRNA/mRNA comparative studies to clarify the cellular and molecular bases of MB origin and maintenance.

Genovesi et al. (2011) assessed miRNA expression in primary human MB specimens, MB cell lines, and, notably, in CD133^+^ NSCs compared to CD133^–^ NPCs. Approximately 30 miRNAs were differentially expressed in primary MB samples compared to CD133^+^ NSCs, many of the upregulated or downregulated species mapping to identical chromosomal regions. By integrating sample-matched miRNA expression profiles with mRNA gene expression data, several putative regulatory networks in MB were identified. An enrichment analysis focused on deregulated mRNA targets revealed overrepresentation of pathways associated with neuronal migration and NS development or with cell proliferation and programmed cell death. The most significantly downregulated miRNA, miR-935, was deeply analyzed, and several putative anticorrelated targets were validated in MB cells.

Along this line, miRNA and mRNA expression in MBSCs derived from *Ptch*^+/–^ mice (SHH-MB, Po et al., 2010) was compared to equivalent profiles from cerebellar NSCs (Po et al., 2018). Medulloblastoma SCs were characterized by 35 upregulated and 133 downregulated miRNAs. The intersection between the gene pathways enriched in the SHH-MBSC miRNAome and transcriptome revealed common gene networks (such as PI3k-Akt pathway and protein processing in endoplasmic reticulum pathway), including putative targets of the differentially expressed miRNAs, reported to play a role in cancer cell growth and maintenance.

MicroRNA and mRNA expression profiling were also paralleled in murine highly tumorigenic MBSCs (from *Ptch*^+/–^; *p53*^–/–^ mice) and low-tumorigenic CSCs (*Ptch*^+/–^; *p53*^+/+^) or normal NSCs (Hemmesi et al., 2015). The expression of miR-135a was inversely correlated with the tumor-initiating potential of MBSCs and with the expression of its target Arhgef6, a factor implicated in the formation of focal adhesion structures essential for cell motility (Rosenberger et al., 2005; Rosenberger and Kutsche, 2006). MiR-135a restoration in MBSCs inhibits tumor progression *in vivo*, and its expression was significantly downregulated in all the histological variants of human MBs.

Taking advantage of analogous model systems, Tanno et al. (2016) explored the miRNome alterations in radiation-induced MB tumorigenesis. MicroRNA expression analysis by NGS in *ex vivo* unirradiated or radiation-treated WT and *Ptch*^+/–^ GNPCs identified a subset of miRNAs controlling different biological functions, as inferred by mRNA network analysis. MicroRNAs were also proposed as putative epigenetic transgenerational messengers responsible for MB susceptibility in the progeny of irradiated male mice (Paris et al., 2015).

Altogether, these and other studies (Catanzaro et al., 2016) confirm the potential of integrated miRNA/mRNA expression analyses to provide insights into MB biology and to identify candidate genes with a functional role in MB progression.

## MiRna-Mediated Gene Pathways in MB

**FIGURE 3:**
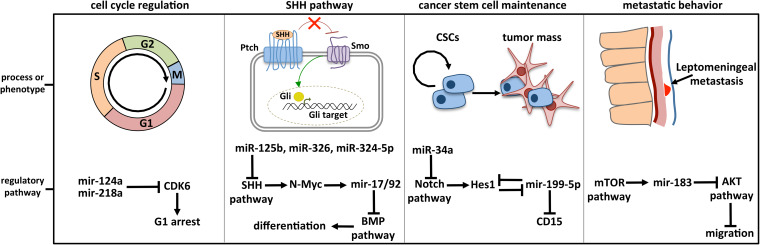
Deeply characterized cellular processes affected by miRNAs in MB. The brain-enriched miR-124a and miR-218a participate in cell cycle control by modulating the expression of CDK6, the cyclin-dependent kinase 6 crucially implicated in cell cycle progression. MiR-125b, miR-326, miR-324-5p, and MiR-17/92 cluster is included in the regulatory axis between the SHH and the BMP signaling, participating in the cell choice between proliferation and differentiation. MiR-125b, miR-326, and miR-324-5p guide the SHH pathway, modulating Smo and Gli1, respectively activator and effector of the cascade. MiR-199-5p is involved in CSC maintenance by modulating the CSC marker CD15 and Hes1, the main Notch downstream effector with which a negative regulative feedback loop is established. Notch signaling is also controlled by miR-34a, at the level of the ligand Dll1. The retinal miR-183 is part of the regulative axis between mTOR pathway, from which it is activated, and AKT pathway involved in cell migration.

Dozens of miRNAs have been specifically linked to MB and already largely reviewed. Four historically relevant and deeply characterized examples of miRNA-regulated molecular pathways in MB are detailed below (Figure 3).

### MiR-124 and Cell Cycle Regulation

MiR-124 is a highly conserved and brain-enriched miRNA, whose role in neural development has been extensively described (Cheng et al., 2009; Yoo et al., 2011). It was the first miRNA linked to MB pathogenesis through targeted analyses.

The observation that overexpression in MB of the adverse prognostic marker Cdk6 (Mendrzyk et al., 2005) cannot be completely explained through its genomic amplification raised the hypothesis of a miRNA-based control of its levels. Due to its preferential expression in differentiating and mature neurons (Gao, 2010), its potential binding to *Cdk6* 3′ UTR and its significant downregulation in both MB cell lines and primary cells (Pierson et al., 2008), Pierson and colleagues focused their attention on miR-124. It was found to be downregulated in patient tumor samples, where miR-124 and *Cdk6* expression levels were inversely correlated. Mir-124 was demonstrated to target *Cdk6* gene and to impact on cell growth when re-expressed in MB. These findings enforced the idea that miR-124 may play a role in MB pathogenesis as a tumor suppressor, controlling *Cdk6* that is crucially implicated in cell proliferation and differentiation (Grossel and Hinds, 2006). Consistently, low levels of miR-124 may explain why MB cells have a less differentiated phenotype than the adult cerebellar tissues. The regulatory axis between mir-124 and *Cdk6* was substantially corroborated in 2013, by Silber et al. (2013). They showed: (1) an opposite trend of expression between the two transcripts in primary MBs; (2) a Cdk6-dependent cell cycle arrest and inhibition of MB cell proliferation through miR-124 ectopic expression and (3) MB growth arrest *in vivo* by intracerebellar or subcutaneous transplantation of miR-124 over-expressing cells in immunosuppressed mice.

Independent research lines further strengthen the idea of miR-124 as a tumor suppressor and cell cycle regulator in MB (Li et al., 2009a; Tenga et al., 2016). Overall, these studies demonstrate a deregulation of miR-124 in brain tumor tissues and cells and suggest the possibility that its altered expression in differentiating NPCs may contribute to brain malignancies. This link is underscored by the finding that REST, the global transcriptional repressor of neuronal differentiation, is one of the most relevant regulators of miR-124 (Conaco et al., 2006). Its abnormal expression in cerebellar NPCs causes the arrest of neuronal differentiation *in vitro* (Su et al., 2004) and the formation of histologically MB-like tumors in implanted mice (Su et al., 2006).

Subsequently, a role for miR-218 in the control of cell cycle in MB was also described, as an additional modulator of *Cdk6* expression (Venkataraman et al., 2013). Consistently with the findings that: (1) Dicer supports proper cerebellar development (Constantin et al., 2013) and curbs MB formation (Zindy et al., 2015) and (2) its ablation impairs the expression of cell cycle regulator genes in MB (Liu et al., 2017), a plethora of miRNAs were described as modulators of cell homeostasis in MB. They mainly affect cell proliferation, differentiation, and apoptosis (Li K. K. et al., 2013; Li et al., 2015; Jin et al., 2014; Xu et al., 2014; Pal and Greene, 2015; Panwalkar et al., 2015; Salm et al., 2015; Senfter et al., 2019; Yang et al., 2019), as well as senescence (Venkataraman et al., 2010) and autophagy (Singh et al., 2017).

### MiR-17/92 Cluster and the SHH Pathway

The miR-17/92 polycistron was one of the first miRNA clusters to be validated as oncogenic (He et al., 2005). Its implication in MB was discovered in 2009 (Uziel et al., 2009), upon deep sequencing and comparative expression analyses of both cerebellar tissues and purified GNPCs from wild-type and MB mouse models. Within the 26 identified miRNAs, putatively acting as oncogenic miRNAs due to lower expression in wild-type samples, as many as nine species were encoded by the miR-17/92 cluster and its paralogues. Upregulated miR-17/92 cluster expression was also confirmed in human MB showing an activated SHH pathway, which suggested a correlation between SHH signaling and miR-17/92 levels.

The association between miR-17/92 and MB was assessed in parallel by Northcott et al. (2009), through single-nucleotide polymorphism arrays aimed to define recurrent copy number aberrations. They identified both amplification and higher expression of miR-17/92 locus in SHH-MB samples compared to normal tissues. Functionally, this study demonstrated that MycN, a TF known to regulate miR-17/92 in other systems together with Myc (O’Donnell et al., 2005; Schulte et al., 2008), was abundantly expressed in tumors with high levels of miR-17/92, and it was able to drive miR-17/92 transcription in GNPCs. This finding, together with the concepts that *MycN* is a downstream target of SHH signaling and drives GNPC proliferation (Kenney et al., 2003; Oliver et al., 2003), shed new light on the regulatory axis through which aberrant SHH signaling drives tumorigenic pathway in MB. Phenotypically, enforced expression of the miR-17/92 cluster in purified primary mouse GNPCs increased the penetrance and accelerated the development of tumors in orthotopically transplanted mice (Uziel et al., 2009). It is noteworthy that this occurred specifically in *Ptch1*^+/–^ GNPCs, where the SHH cascade is aberrantly activated (Evangelista et al., 2006), which definitively demonstrates the functional link between the SHH pathway and the miRNA cluster. Indeed, even though dispensable for cerebella development, miR-17/92 cluster is necessary for SHH-MB tumor formation (Zindy et al., 2014). To clarify the underlying control circuitry, reverse genetics approaches were undertaken (Murphy et al., 2013). LNA-anti-miR–mediated miR-17 and miR-19a silencing in primary cells from SHH-MB led to suppression of cell growth *in vitro* due to cell cycle arrest and to inhibition of secondary tumor progression in allograft mice. MiR-17 silencing also reduced the progression of tumors derived from intracranial transplants and increased survival. These effects may be explained by the finding that Brmp2, a member of the bone morphogenetic protein (BMP) signaling pathway that induces the differentiation of SHH-MB (Zhao et al., 2008), is a target of miR-17 and miR-19.

### MiRNAs and MB Cancer Stem Cells

The Notch signaling plays an essential role in the regulation of cellular processes during embryonic and postnatal development (Artavanis-Tsakonas et al., 1999). In the cerebellum, it regulates the differentiation of GNPCs (Solecki et al., 2001). Notch cascade is also involved in the maintenance of MBSCs. Consistently, pathway components such as the receptor Notch2 and the TF Hes1, the main Notch downstream effector, are deregulated in MB (Fan et al., 2004). *In silico* inspections of miRNA databases predicted miR-199-5p as a putative modulator of *Hes1*, and this regulative axis was confirmed by reverse-genetics approaches (Garzia et al., 2009). Cellular and molecular assays demonstrated that the stable expression of miR-199-5p in several MB cell lines reduced their proliferation rates through cell-cycle alterations, increased the expression of differentiation markers, and impaired clonogenic potential. Interestingly, clones stably expressing miR-199-5p were depleted of a CD133^+^ cancer stem-like cell side-population, whereas restoration of *Hes1* expression partially rescued cell cycle block, decreased cell differentiation, and recovered side-population cells. This demonstrates that *Hes1* downregulation was directly correlated with these phenotypes. The potentiality of miR-199b-5p as a tumor suppressor was validated *in vivo*, in xenografts derived from stably expressing miR-199-5p clones. On the other hand, miR-199b-5p was downregulated in metastatic MB samples where Hes1 protein is upregulated, thus confirming their participation in the same regulatory module.

The molecular circuit underlying miR-199-5p expression was deeply unraveled by Zollo’s group, who revealed an exciting negative feedback loop between the repressor Hes1 and the miRNA (Andolfo et al., 2012). *Hes1* inhibition or silencing caused a significant increase of miR-199-5p, mediated by the presence of Hes1 binding sites on miR-199b-5p promoter. Furthermore, an additional layer of regulation was highlighted, dependent on the methylation status of a CpG island in the region upstream to miR-199-5p promoter. Functionally, the tumor-propagating cell marker CD15 was demonstrated to be a direct target of miR-199-5p, which also alters the expression and phosphorylation of the major proteins of AKT and ERK networks, involved in cancer metastasis and cancer stem-cell maintenance.

The control of Notch pathway in MB is also contributed by other miRNAs, such as the key regulator of neuronal differentiation miR-9 (Fiaschetti et al., 2014) and the p53-dependent miR-34a (He et al., 2007). MiR-34a was deeply analyzed in MB. It was found to affect Notch signaling as a negative regulator of the major ligand of Notch receptor, Delta-like 1 (Dll1) (de Antonellis et al., 2011), which contributes to the maintenance of the undifferentiated state of neural progenitors in CNS (Kawaguchi et al., 2008). MiR-34a–sustained expression induced apoptosis and inhibited proliferation of CD15^+^/CD133^+^ MBSCs *in vitro*, promoting neural differentiation. *In vivo*, miR-34a inhibits tumor growth in orthotopically and heterotopically transplanted nude mice through inhibition of AKT/PI3K/PTEN signaling, which is responsible for the maintenance and propagation of this cell population.

The pleiotropic role of miR-34 as a tumor suppressor is paradigmatic. In MB, it potently inhibits c-Met, a tyrosine kinase receptor activating a range of signaling pathways in development and cancer (Li et al., 2005), and suppresses numerous malignancy parameters *in vitro* (Li et al., 2009b). Its deficiency in MB mouse models accelerates the incidence and timing of tumor formation *in vivo* (Thor et al., 2015), possibly affecting multiple targets, among which MycN, frequently amplified in SHH-MB and known to drive GNPC proliferation during medulloblastomagenesis. A body of evidence indicates that miR-34a may represent a potential therapeutic agent in MB, by modulating at multiple levels p53 tumor suppressor pathway (Fan et al., 2014) and conferring sensitivity to chemotherapeutic treatments. In particular, the identification of targets, among which miRNAs (Lee et al., 2014; Abdelfattah et al., 2018; Ray et al., 2018), serving as effective and low neurotoxic adjuvants to treat MB, is of paramount importance. This is due to the high risk of MB relapses and to the severity of side effects related to therapeutic regimens. The implication of miRNAs in MBSC biology (Lee et al., 2014; Kaid et al., 2015; Besharat et al., 2018) may help for this search.

### MiRNAs and MB Metastases

The spread of cancerous cells to the spinal and intracranial leptomeninges (leptomeningeal dissemination) is the principal hallmark of MB unfavorable outcome, due to fatal tumor recurrence (Rossi et al., 2008). This explains the deep interest toward the comprehension of the molecular mechanisms responsible for MB metastatic behavior. A miRNA screening of 32 MB samples indicated that the retinal miRNAs miR-182, miR-183, and miR-96, members of the oncogenic miR-183/96/182 cluster (Xu et al., 2007), were significantly overexpressed in non-SHH-MBs (Bai et al., 2012). Two candidates, miR-182 and miR-183, were further phenotypically characterized, showing an increased migratory effect upon overexpression in MB cells and a decreased motility propensity upon their knockdown. *In vivo*, orthotopic xenograft experiments revealed that miR-182 overexpressing tumors extensively infiltrated in the surrounding normal tissue compared to control samples and, importantly, showed local leptomeningeal metastases.

An independent study further investigated the function of miR-183/96/182 cluster in the maintenance, survival, and dissemination of tumor cells in the aggressive *Myc*-amplified subgroup, where the miRNA cluster was upregulated (Bai et al., 2012). LNA-mediated knockdown of miRNA cluster components in MB cell lines revealed decreased cell proliferation and viability, cell cycle arrest, and increased senescence and expression of apoptotic markers. Gene expression and proteomic profiling upon miR-183/96/182 knockdown allowed identifying enrichment of gene sets associated with several biological pathways. Authors functionally linked the increase of invasion/migration signature with the enrichment of genes associated with PI3K/AKT/mTOR pathways (Irie et al., 2005) and demonstrated that mTOR is required for both miR-183 cluster activation and metastatic activity in MB. MiR-183 cluster was recently described as highly expressed also in mouse models for SHH-MB subgroup inactivated for the tumor suppressor *Pten*, which alters tumor histology and accelerates tumorigenesis. In this condition, miR-183/96/182 cluster promotes MB development by controlling GNPC proliferation (Zhang et al., 2013).

Microarray profiling of seeding *versus* non-seeding MBs revealed 12 differentially expressed miRNAs (Yang et al., 2015), among which miR-192, known to regulate epithelial–mesenchymal transition (Wang et al., 2010), a transdifferentiation process favoring cancer cells migration from the primary tumor site, invasion of surrounding tissues, and eventually metastasis generation (Brabletz et al., 2005). MiR-192 inhibited the anchoring ability of MB cells *in vitro* and *in vivo* by targeting several integrins and integrin-related proteins, central regulators of focal adhesion dynamics. Therefore, miR-192 downregulation in MB may promote leptomeningeal dissemination through cancer cell adhesion to secondary sites.

MiR-21 is associated to the metastatic behavior of several neoplasms, including brain tumors (Chan et al., 2005). Its expression levels were found upregulated in 29 MB samples and inversely correlated to the levels of its target *Pdcd4*, a gene suppressing metastasis in human cancer cells through inhibition of invasion-related proteins (Grunder et al., 2011). In MB, this decreased the activity of factors implicated in cell migration/invasion (such as AKT, c-JUN, and ERK), or in solid cancer dissemination/metastases, such as Timp-2, uPAR, E-cadherin, and integrin. Consistently, inhibition of miR-21 reduced MB cell motility and invasiveness.

Besides the instances collected above, additional miRNAs are potentially linked to MB dissemination, based on their involvement in cancer cell migration, invasivity, and metastatic behavior (Gao et al., 2015; Yogi et al., 2015; Pan et al., 2017).

## LncRNAs in MB, From Past to Present

Similarly to miRNAs, a large number of lncRNAs have been characterized as potential oncogenes or oncosuppressors in cancer, including CNS tumors (Table 1). Long ncRNAs are considered as promising biomarkers and therapeutic targets in oncology; nonetheless, our comprehension of their involvement in MB is still largely incomplete.

**TABLE 1:** Summary of the features and function of lncRNAs, circRNAs, and snRNAs involved in MB.

Class	Name	Alteration	System	Observed phenotypes	Mechanism/target	References
**Long ncRNA**						
Oncogene	ANRIL	Genetic variant	Primary tumors	Predisposition to MB	/	Chen et al., 2018
	ANRIL	Upregulation	MB cells	Cell viability migration, apoptosis	Decoy of miR-323; increase of BRI3; induction of MAPK, AKT, WNT pathways	Zhang et al., 2017a
	PVT1	Rearrangement	Group 3	Cell proliferation	miR-1204 upregulation	Northcott et al., 2012c
	Linc-Ned125	Upregulation	Group 4	Cell proliferation, migration invasivity	Decoy of miR-19a-3p, miR19b-3p, mir-106a5p; increase of Group 4 driver genes	Laneve et al., 2017
	CCAT1	Upregulation	Primary tumors	Cell proliferation, migration invasivity, tumor development	Post-translational modification of AKT pathway	Gao et al., 2018b
	LOXL1-AS1	Upregulation	Primary tumors	Cell proliferation, apoptosis, clonogenic potential, cell cycle, migration; EM transition; tumor size and weight	Activation of PI3K/AKT pathway	Gao et al., 2018a
	TP73-AS1	Upregulation	SHH	Cell viability, proliferation migration; tumor survival, growth, aggressiveness	Decoy of miR-494-3p upregulation of EIF5A2	Li et al., 2019; Varon et al., 2019
	HOTAIR	Upregulation	Primary tumors	Cell viability, colony formation, apoptosis, migration and invasion, tumor growth	Decoy of miR-1 and miR-206; upregulation of YY1	Zhang et al., 2020
	UCA1	Upregulation	Primary tumors	Cell cycle, migration, proliferation, aggregation, and apoptosis		Zhengyuan et al., 2017
	CRNDE	Upregulation	Primary tumors	Cell viability, proliferation, colony formation, apoptosis, migration, invasion, chemosensitivity tumor growth	Decoy of miR-29c-3p	[Bibr B217][Bibr B222]
	SPRY4-IT1	Upregulation	MB cells	Cell proliferation, invasion, migration		Shi et al., 2017
	EVF-2	Upregulation	Primary tumors			Bonfim-Silva et al., 2013
Oncosuppressor	Nkx2-2as	Downregulation	SHH	Cell proliferation, apoptosis invasion, colony formation tumor growth	Decoy of miR-103a/107 and miR-548; downregulation of Btg2, Lats1, Lats2	Zhang et al., 2018
	HOTAIR	Downregulation	Primary tumors		Upregulation of Hoxd8 and Hoxd10	Chakravadhanula et al., 2014
circRNA	Circ-SKA3 circ-DTL	Upregulation	Primary tumors	Cell proliferation, migration, invasion	Upregulation of host transcripts	Lv et al., 2018
snRNAs	U1snRNA	Mutation	SHH	Patient survival	Dysregulation of oncogene and oncosuppressor splicing	Suzuki et al., 2019

The first lncRNA analyzed in MB was H19 (Albrecht et al., 1996). H19 gene was already known to code for a small spliced and polyadenylated RNA not containing any long, conserved ORF. Even though its function was not clarified yet, it was known that H19 transfection in cell lines suppressed tumorigenesis (Hao et al., 1993) and that, due to genomic imprinting of the paternal promoter, H19 was expressed from the maternal (non-imprinted) allele (Zhang et al., 1993). Because biallelic gene expression [i.e., loss of imprinting (LOI)] of *IGF2*, a maternally imprinted gene regulated by H19 (Leighton et al., 1995), occurs in tumors (Ogawa et al., 1993; Rainier et al., 1993), Albrecht et al. (1996) examined, by allele-specific RT-PCR, IGF2 and H19 imprinting in fetal cerebella, MB samples, and cell lines. They found only a partial H19 LOI in 50% of MBs analyzed, independently of *IGF2* imprinting, indicating that, in MB, expression and imprinting of the two genes are individually controlled.

Over years, high-throughput investigations dramatically entered the scene in molecular oncology. Even though -omic and integrative approaches provide a valuable source of data for identifying lncRNAs and may potentially reveal intriguing aspects of MB, less than 20 lncRNAs have been associated to this malignancy to date. In some cases, MB lncRNAs emerged by *de novo* sequencing experiments or dataset interrogations (see PVT1 and TP73-AS1, next section), whereas the vast majority of lncRNAs characterized in MB derived from previous studies, carried out in different pathological model systems. Nevertheless, only for a few of them the molecular mechanisms have been thoroughly elucidated. Based on the idea that similar mechanisms of action underlie different diseases with overlapping phenotype associations, Xu et al. (2017), through construction of gene and lncRNA coexpression networks, generated a computational approach to systematically prioritize and identify candidate disease risk lncRNAs in 14 cancer types, including MB. This method may help associating identified lncRNA-based mechanisms to human cancers. Along the same line, preliminary reports indicate that genome-wide data reanalyses of lncRNA expression profiles in MB may be useful for identification of lncRNA signatures with a diagnostic, prognostic, or functional value (Joshi and Perera, 2019 preliminary report).

### LncRNAs AS MB ONCOGENES

PVT1 was the first lncRNA gene identified in human cancer translocations (Graham and Adams, 1986; Shtivelman et al., 1989). Its role in cancer is still debated: PVT1 has an oncogenic potential, by stabilizing Myc protein (Tseng et al., 2014), whereas its promoter functions as a tumor suppressor DNA element that limits *Myc* oncogene expression and activity (Cho et al., 2018). PVT1 locus has been suggested to be a fragile site (Colombo et al., 2015), and its gene is frequently amplified together with *Myc* in group 3 MB (Northcott et al., 2012c). Accordingly, RNA-Seq of group 3 MB revealed a gene fusion involving the 5′ end of PVT1 and the coding region of *Myc*. This represents the first identified MB group 3–specific gene fusion, which occurs through events of chromosome fragmentation due to erroneous DNA repair events (chromothripsis).

PVT1 is the host transcript of 4 putative oncogenic miRNAs (miR-1204, miR-1205, miR-1206, and miR-1207, Beck-Engeser et al., 2008). MiR-1204, whose sequence is comprised in the rearranged genomic region, was upregulated in group 3 PVT1-*Myc* fusion(+) tumors, where it contributes to the malignant phenotype. Indeed, inhibition of miR-1204 reduced cell proliferation. In turn, *Myc* knockdown resulted in diminished expression of miR-1204, which is in line with the observation that the PVT1 promoter contains non-canonical Myc responsive E-boxes. These observations suggest a positive feedback loop through which Myc reinforces its own expression and increases the levels of the pathological miR-1024 in PVT1-*Myc* fusion(+) tumors (Northcott et al., 2012c).

A different mechanism altering miRNA activity in a lncRNA-dependent manner is the miRNA sequestration (decoy), which emerged as a relevant pathogenic pathway in several cancers (Tay et al., 2014). In this case, lncRNAs function as competing endogenous RNAs (ceRNA), able to sponge miRNAs by base annealing, inhibiting their activity and causing translational derepression of their target mRNAs.

**FIGURE 4:**
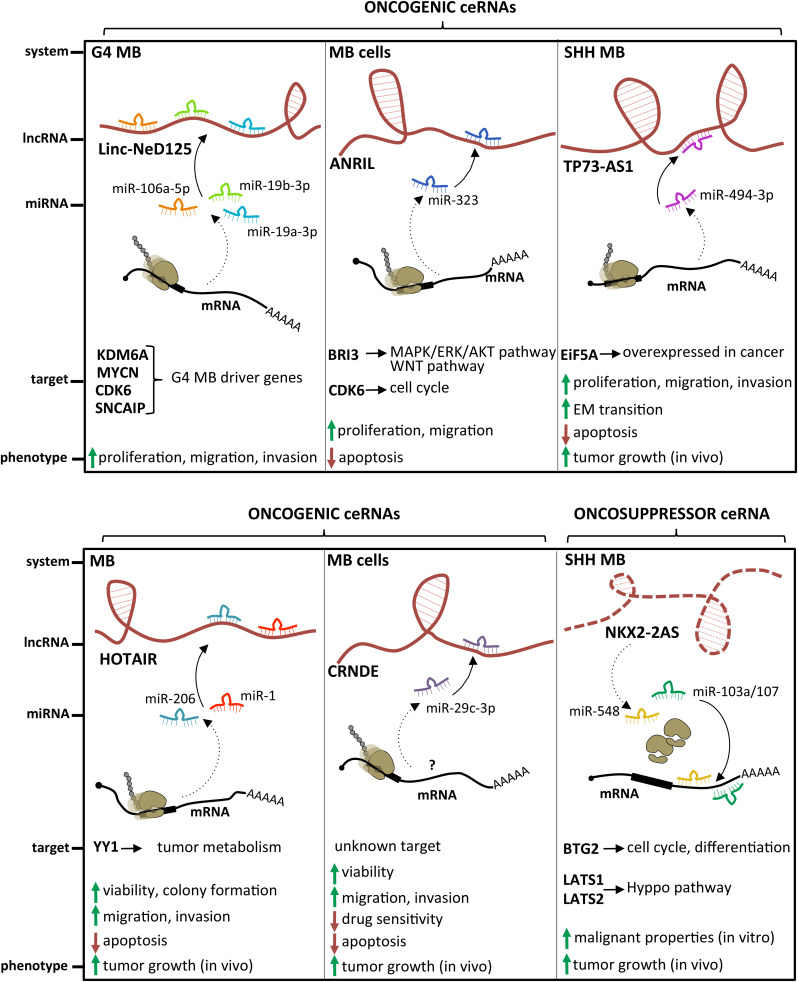
State of the art on lncRNAs functioning as oncogenes or oncosuppressors through their competing endogenous RNA (ceRNA) activity. A handful of lncRNAs, such as Linc-NeD125, ANRIL, TP73-AS1, HOTAIR, and CRNDE, may function as oncogenes by sponging specific miRNAs and leading to derepression of their target genes. Differently, only NKX2-2AS has been described as a lncRNA endowed with oncosuppressor activity.

Several examples of lncRNAs acting as miRNA sponges have been described in MB (Figure 4). The lncRNA linc-NeD125 was the first ceRNA described in brain tumors. This transcript was identified in NB-derived cells, as the primary transcript for miR-125-b1 (Bevilacqua et al., 2015), a neuronal miRNA (Smirnova et al., 2005) involved in neural differentiation and function (Boissart et al., 2012; Akerblom et al., 2014) and in cancer cell proliferation (Laneve et al., 2007; Ferretti et al., 2009). In an *in vitro* neuronal differentiation model, linc-NeD125 was shown to be induced upon differentiation stimulus and to act synergistically to miR-125 to promote neuronal differentiation (Bevilacqua et al., 2015). Linc-NeD125 was then revealed as a novel potential biomarker and therapeutic target in MB (Laneve et al., 2017). By RT-PCR analyses, linc-NeD125 was found to be highly expressed in group 4 MB, whereas RNA pull-down and CLIP experiments highlighted its capacity to recruit a pool of miRNAs (miR-19a-3p, miR-19b-3p, and miR-106a-5p), able to alter MB cell tumorigenic properties. Consistently, these miRNAs were validated as regulators of at least four group 4 MB driver genes (*Kdm6A, MycN, Cdk6*, and *Sncaip*). Importantly, alterations of linc-NeD125 expression impinge on the same gene network and biological phenotypes *in vitro*, pointing to this transcript as a non-coding contributor of group 4 MB tumorigenesis or cancer cell maintenance.

An additional transcript acting as a ceRNA in MB is the lncRNA ANRIL, already implicated in several malignancies where it functions as a *cis*-acting epigenetic silencer of the tumor suppressor *Ink4B* (Yap et al., 2010; Kotake et al., 2011). Its levels are enhanced in MB cells (Zhang et al., 2017a), and its repression affects the expression of multiple apoptotic factors, such as *Bcl-2*, *Bax*, cleaved/procaspase-3, and cleaved/procaspase-9, and impacts on cell viability and migration. Mechanistically, ANRIL is able to sequester miR-323, a neuronal miRNA acting as a tumor suppressor in brain cancer (Lavon et al., 2010; Qiu et al., 2013). Notably, the ANRIL/miR-323 regulatory module controls the expression of the brain-specific factor *Bri3* (Vidal et al., 2001), which induces MB-associated pathways, such as MAPK, AKT, and WNT, through both posttranscriptional and posttranslational mechanisms.

Exploiting genome-wide association studies on brain tumors, which have identified multiple disease susceptibility loci (Kinnersley et al., 2015), Chen et al. (2018) tested the hypothesis that some glioma-risk single-nucleotide polymorphisms may contribute to MB predisposition. They found that the rs2157719 T > C genetic variant of ANRIL was significantly associated with an increased MB risk in the Chinese population, supporting the idea that brain tumors might partially share genetic risk factors and etiological pathways.

A paradigmatic example of oncogenic lncRNA is CCAT1 (Nissan et al., 2012), linked to several tumors ranging from hepatocellular carcinoma to breast cancer, where it stimulates malignant phenotypes such as proliferation, invasion, migration, and chemoresistance (Chen et al., 2016; Ma et al., 2017; Zhang et al., 2017b). CCAT1 is overexpressed in MB primary tumors and cell lines (Gao et al., 2018b). *In vitro*, its downregulation causes the reduction of MB cell proliferation, migration, and invasion, whereas its depletion *in vivo* reduces the development of subcutaneously transplanted tumors. Similarly to ANRIL described above, also CCAT1 may play its oncogenic function by altering posttranslational modifications of the tumorigenic MAPK pathway components. However, parallel studies revealed its activity as a miRNA decoy in different tumor model systems, raising the hypothesis of multiple modes of action.

Differently from the previous cases, the tumorigenic role of the LOXL1-antisense RNA (LOXL1-AS1) was addresses for the first time in MB (Gao et al., 2018a). Its expression was higher in 36 out of 50 MB tissues, compared to paired non-cancerous resections. LOXL1-AS1 depletion in two MB cell lines inhibited cell proliferation and clonogenic potential, arrested cell cycle progression at the S phase, slowed cell migration, promoted cell apoptosis rate, and reverted epithelial–mesenchymal transition. Furthermore, LOXL1-AS1 knockdown in xenograft mouse models caused a significant reduction of tumor size and weight. The decreased level of PI3K and AKT phosphorylation observed upon LOXL1-AS1 RNAi suggested that LOXL1-AS1 may activate the PI3K/AKT pathway in MB.

The latest oncogenic lncRNA characterized in MB was TP73-AS1 (Varon et al., 2019), a transcript antisense to p73 mRNA that encodes for a member of the p53 TF family (Dötsch et al., 2010). p73 is implicated in brain development (Niklison-Chirou et al., 2013) and cancer (Flores et al., 2005), including MB (Niklison-Chirou et al., 2017). Similarly to was what observed in a subgroup of glioblastoma with better diagnosis (Sturm et al., 2012), comparative expression analyses of TP73-AS1, using the R2 platform^1^ and the Cavalli cohort (Cavalli et al., 2017), revealed its upregulation in SHH-MB, where TP73-AS1 promoter is significantly hypomethylated (according to Schwalbe dataset for CpG island methylation, Schwalbe et al., 2017). In SHH-MB cells, TP73-AS1 supports cell survival and division, proliferation, viability, and migration, irrespective from TP53 status (wild type or mutant). Supporting the genetic independency of TP73-AS1 and P53 family, TP73 knockdown did not impact the levels of TP73-AS1 and *vice versa*. *In vivo* experiments indicate that TP73-AS1 supports MB tumor survival, growth, and aggressiveness, both in cancer tissues and in explanted cell cultures. The mechanism of action of TP73-AS1 as a miRNA decoy was clarified very recently (Li et al., 2019). TP73-AS1 was demonstrated to promote MB growth *in vitro* and *in vivo* and to modulate the expression of the eukaryotic translation initiation factor *EIF5A2*, an oncogene upregulated in several cancers including MB (Yang et al., 2019) by sponging miR-494-3p, previously reported as a putative oncogenic miRNA (Lin et al., 2018).

The role of the prototypical lncRNA HOTAIR (Rinn et al., 2007) in MB is still controversial. One study described it as underexpressed in MB samples, where its target genes *Hoxd8* and *Hoxd10* were upregulated (Chakravadhanula et al., 2014). Another report (Zhang et al., 2020) found it expressed at high levels in MB tissues and cell lines, where it interacts with and modulates miR-1 and miR-206, which in turn directly target the oncogenic TF YY1. Rescue assays in MB confirmed the occurrence of this further ceRNA network, which controls cell viability, ability to form colonies, apoptosis, migration, and invasion *in vitro*, as well as tumor growth in xenografted mice.

### LncRNAs as MB Tumor Suppressors

Only one lncRNA has been described as an oncosuppressor in MB. Microarray analysis performed in SHH-MB–derived cells and GNPCs indicated the lncRNA Nkx2-2as as the gene showing the highest degree of downregulation, which is dependent on aberrant SHH signaling (Zhang et al., 2018). Specifically, Nkx2-2as expression in SHH-MB cells is suppressed by the repressor Foxd1, which is induced by the SHH-responsive TF Gli2. Gli2/Foxd1-mediated Nkx2-2as downregulation contributes to the pathogenesis of SHH subgroup. Nkx2-2as ectopic expression in MB cell lines impaired cell growth, colony formation, and invasion, while increasing apoptosis; its knockdown in CGNPs promoted their proliferation. Consistently with *in vitro* results, Nkx2-2as overexpression delayed tumor growth in xenograft mouse models. Rather than through its antisense transcript Nkx2-2 (Tochitani and Hayashizaki, 2008), Nkx2-2as functions as miRNA decoy in MB. It sequesters the oncogenic miRNAs miR-103a/107 and miR-548, whose cellular targets, the cell cycle factor *Btg2* and the Hippo pathway regulators *Lats1* and *Lats2*, function as tumor suppressors in MB (Farioli-Vecchioli et al., 2012; Rao et al., 2016).

### Other LncRNAs in MB

A handful of studies suggest a role in MB for some lncRNAs whose molecular mechanism has not yet been entirely clarified. This is the case for the lncRNAs UCA1 (Xiao et al., 2016), which is upregulated in MB samples. It has been demonstrated that it is able to alter MB cell properties, as cell cycle progression, migration, proliferation, aggregation, and apoptosis when downregulated *in vitro* (Zhengyuan et al., 2017). Similar findings have been described for SPRY4-IT1 (Shi et al., 2017) and for CRNDE, a lncRNA already characterized in MB (Song et al., 2016) and other tumors. A molecular interaction was found between CRNDE and miR-29c-3p, whose altered expression affected tumor growth *in vivo* and tumorigenic cell features *in vitro* (Sun et al., 2020). Interestingly, expression studies in MB cell lines exposed to cisplatin treatment revealed that elevated levels of CRNDE are associated with resistance to chemotherapeutics and, consistently, functional studies demonstrated an increased cell chemosensitivity upon miR-29c-3p overexpression. The molecular target of this regulative axis is still unknown.

Finally, one report indicates that the lncRNA EVF-2 is overexpressed in MB cell lines and tissues, compared to control samples (Bonfim-Silva et al., 2013).

## Other Classes of ncRNAs in MB: circRNAs, eRNAs, and snRNAs

Brand-new molecular classes of ncRNAs have been discovered in the last few years, as the results of deep explorations of genome transcriptional outputs.

Circular RNAs are covalently closed continuous loops instead of canonical linear forms. They derive from the joining of a 5′ splice site and a 3′ splice site as the result of back splicing. They are characterized by the lack of 5′ cap and 3′ tail and, due to their unique structure, by high stability and relatively long half-life. Previously considered as aberrant by-products of linear transcript splicing, they are becoming the focus of research interest for their relative abundance, their conservation and tissue specificity, and their enrichment in NS. Recent studies have highlighted the implication of these still enigmatic transcripts in processes as diverse as differentiation and development and their activities as epigenetic and transcriptional regulators, miRNA sponges, and RNA-binding protein decoys (Memczak et al., 2013; Zhang et al., 2016; Kristensen et al., 2019).

As a consequence of their pervasive activity, altered expression of circRNAs occurs in several pathologies, including brain cancers (Bhan et al., 2017). To investigate the expression profile and function of circRNAs in MB, NGS was performed on RNA from 4 human MBs, revealing more than 15,000 distinct circRNAs with more than 1 back-spliced read (Lv et al., 2018). Notably, 36 differentially expressed circRNAs were identified in MB *versus* normal tissues, most of them being downregulated. Two upregulated candidates, circ-SKA3 and circ-DTL (Table 1), putatively acting as circular oncogenes, were functionally characterized. Loss-of-function assays revealed that the two species were able to regulate the expression of their host transcripts, previously demonstrated to be important in cancer progression (Kobayashi et al., 2015; Chuang et al., 2016). Parallel rescue experiments with mRNA-overexpressing plasmids indicate that circRNA-dependent dysregulation of the linear counterpart impacts on MB cell proliferation, migration capacity, and invasion ability *in vitro*.

Enhancer RNAs are *cis*-acting transcripts synthetized at active enhancers (De Santa et al., 2010; Kim et al., 2010). They contribute to the transcriptional regulation of target genes by recruiting key TFs or chromatin-associated complexes and by forming high-dimensional DNA structures between enhancer, super-enhancers (clustered enhancers), and promoters or by stimulating RNA polymerase II elongation (de Lara et al., 2019). Although the studies of their biological roles are still at infancy, the eRNA aberrant regulation seems to be closely related to tumorigenesis (Li W. et al., 2013).

Lin et al. (2016) profiled the enhancer landscape in 28 MB primary tumors by ChIP-Seq analysis for H3K27ac and Brd4, which are marks of active enhancers. Through combined analyses aimed at: (1) matching differentially active enhancers and target genes within the same topologically associated domain and (2) clustering spatially colocalized enhancer domains (super-enhancers), authors sought to clarify MB subgroup identities and origin from a novel perspective. Specifically, they reconstructed core regulatory circuitries in MB subgroups, focusing on master transcriptional regulators. They identified novel TFs implicated in MB development and traced their expression along cerebellar formation, which led to propose the deep cerebellar nuclei in the nuclear transitory zone as the putative cells of origin for G4 MB.

Small nuclear RNA (snRNA) implication in splicing dynamics and regulation is a longstanding dogma of gene expression (Lerner et al., 1980). By combining extensive inspections of MB whole-genome sequencing data with allele-specific PCR analyses, Suzuki et al. (2019) recently revealed in a large fraction of SHH-MBs (97% of adult SHHδ cases and 25% of SHHα adolescent cases) a recurrent hotspot mutation of the snRNA U1 gene (Figure 1 and Table 1), which is associated with an extremely poor prognosis when combined with *TP53* mutation. U1 snRNA mutation (r.5A > G) mapped in the highly conserved 5′ splice-site recognition sequence of the snRNA. In line with this finding, computational and experimental approaches indicate that the mutation does affect splicing. Intron-centric alternative splicing analysis proved that mutant U1 snRNA variants display a threefold increase of alternative 5′ cryptic splicing events, whereas complementary exon-centric alternative splicing analysis revealed a higher incidence of cassette exons events and intron retention. Mutant U1-mediated aberrant alternative splicing inactivates tumor-suppressor genes (*Ptch1, Pax5*) and activates oncogenes (*Gli2* and *Ccnd2*) or affects genes linked to SHH pathway (*Pax6*) or to other tumors (*Tox4*). This study reveals how, besides identifying novel species involved in cerebellar cancer, genome-wide explorations with a careful focus on non-coding loci allow better understanding the contributions of well-known non-coding RNAs to MB. Specifically, it indicates that single-nucleotide variants are not limited to proteinogenic genes, reveals the need of prioritized interventions for SHH patients carrying U1 snRNA mutation, highlights the relevance of aberrant posttranscriptional (de)regulation in MB, and suggests opportunities for novel targeted therapeutic approaches.

## Concluding Remarks

In the last decades, our point of view on molecular oncology has tremendously changed, especially thanks to deep studies on RNA biology.

Since 2000s, the progresses in deep sequencing resolution and bioinformatics analyses have enriched our interpretation of the genomic output, providing the notion that mammalian genomes are pervasively transcribed and enlarging the catalog of regulatory transcripts. This promoted the identification of a previously unsuspected layer of gene expression modulation, mediated by ncRNAs. The extensive implication of ncRNAs in cell physiopathology represents one of the foremost revolutions in the recent molecular biology.

At the same time, our comprehension of cancer biology has greatly improved, and MB represents a paradigmatic example of such conceptual advancements. The reclassification of MB in diverse genetic/histological subgroups and subtypes, mainly based on integrative -omics analyses together with the characterization of MB cells of origin, contributes to resolve the conundrum of the tumor intrinsic heterogeneity and to highlight causative developmental aberrations. This propelled further genetic and gene expression analyses aimed at precisely identifying the tumor-driving molecular alterations, as a source for possible biomarkers and targets for therapy. On the other hand, reshaping our notion of MB has triggered, and still requires, the rising of novel *in vitro*, *ex vivo*, and *in vivo* preclinical models, from cell lines to reprogrammed cells, tumor grafts, engineered mice, and, more recently, organoids. Finally, the identification of MBSCs has shed new light on the contribution of cell stemness to tumor maintenance/propagation and to the establishment of malignant phenotypes, such as the resistance to chemoradiotherapeutic treatments and the tendency to dissemination and relapses.

Importantly, the combination of identification of ncRNA-based circuits, as novel theranostic agents, and improved knowledge of MB molecular and cellular biology has favored the development of MB precision oncology.

As largely discussed, short and long regulatory RNAs are broadly engaged in all the aspects of MB and can therefore be considered as powerful diagnostic, prognostic, and therapeutic targets or adjuvants. The possibility to easily detect stable species (miRNAs and circRNAs) in biopsies, body fluids, or preserved samples may support patient stratification, individual diagnosis, and clinical interventions. Furthermore, the function of ncRNAs as oncogenes or oncosuppressors is particularly suitable for translational applications in tumor therapy. This is true both for miRNAs, whose pleiotropic and combinatorial regulatory activity enhances the efficient targeting of complex downstream gene cascades, and for lncRNAs, whose expression is highly tissue-specific and whose function as ribonucleoprotein scaffolds could be blocked through small inhibitory drugs. In addition, the characterization of specific pathological ncRNA-driven gene networks offers novel opportunities for the treatment of diseases implying the alteration of undruggable genes, factors, or pathways, which is a crucial aspect in pediatric tumors.

Despite the potentialities of ncRNA-dependent personalized medicine, no any clinical trial for ncRNA therapeutics is currently ongoing in MB patients. This is mainly due to technical issues, related to the stability of the targeting molecules, and the specificity of delivery. The evolution of reverse genetics approaches supported by modified nucleic acids (for antisense/mimic or RNA silencing strategies), the exploitation of viral carriers–based constructs or gene editing, and the accurate design of novel biomaterials and rationales for nanotechnologies promise to facilitate the administration of targeting species through the plasma membranes and the blood–brain barrier, which remains a major challenge in the field of brain cancer treatment.

## Author Contributions

PL and EC reviewed the literature and wrote the manuscript.

## Conflict of Interest

The authors declare that the research was conducted in the absence of any commercial or financial relationships that could be construed as a potential conflict of interest.

## Footnotes

1 https://hgserver1.amc.nl/cgi-bin/r2/main.cgi
